# Involvement of Skeletal Muscle Gene Regulatory Network in Susceptibility to Wound Infection Following Trauma

**DOI:** 10.1371/journal.pone.0001356

**Published:** 2007-12-26

**Authors:** Yiorgos Apidianakis, Michael N. Mindrinos, Wenzhong Xiao, George P. Tegos, Michail I. Papisov, Michael R. Hamblin, Ronald W. Davis, Ronald G. Tompkins, Laurence G. Rahme

**Affiliations:** 1 Department of Surgery, Harvard Medical School and Massachusetts General Hospital, Boston, Massachusetts, United States of America; 2 Shriner's Burns Institute, Boston, Massachusetts, United States of America; 3 Department of Biochemistry, Stanford University School of Medicine, Stanford, California, United States of America; 4 Department of Dermatology Harvard Medical School and Wellman Center for Photomedicine, Massachusetts General Hospital, Boston, Massachusetts, United States of America; 5 Laboratory of Molecular Bioengineering, Division of Nuclear Medicine, Department of Radiology, Massachusetts General Hospital and Harvard Medical School, Boston, Massachusetts, United States of America; 6 Department of Microbiology and Molecular Genetics, Harvard Medical School, Boston Massachusetts, United States of America; Massachusetts General Hospital, United States of America

## Abstract

Despite recent advances in our understanding the pathophysiology of trauma, the basis of the predisposition of trauma patients to infection remains unclear. A *Drosophila melanogaster/Pseudomonas aeruginosa* injury and infection model was used to identify host genetic components that contribute to the hyper-susceptibility to infection that follows severe trauma. We show that *P. aeruginosa* compromises skeletal muscle gene (SMG) expression at the injury site to promote infection. We demonstrate that activation of SMG structural components is under the control of cJun-N-terminal Kinase (JNK) Kinase, Hemipterous (Hep), and activation of this pathway promotes local resistance to *P. aeruginosa* in flies and mice. Our study links SMG expression and function to increased susceptibility to infection, and suggests that *P. aeruginosa* affects SMG homeostasis locally by restricting SMG expression in injured skeletal muscle tissue. Local potentiation of these host responses, and/or inhibition of their suppression by virulent *P. aeruginosa* cells, could lead to novel therapies that prevent or treat deleterious and potentially fatal infections in severely injured individuals.

## Introduction

Severe injury produces local and systemic deficits that can lead to skeletal muscle wasting and weakness, immune dysfunction, infection, and multiple organ dysfunction syndrome (MODS), with subsequent morbidity and mortality [1]. While immuno-inflammatory responses are induced to mediate defense and orchestrate tissue repair, the mechanism mediating the increased susceptibility to infection following injury remains unresolved. Advances in our understanding of trauma pathophysiology have not yet produced treatments to reduce susceptibility to infection and mortality of injury-induced MODS patients.

In mammals, acute and sustained responses are induced to promote tissue repair following trauma. Tightly regulated immuno-inflammatory responses mediate both the pathophysiology of trauma and post-trauma healing [1], [2]. However, the predisposition of individual patients to infection and other concomitant dysfunctions remains unclear. The observations that injury primarily affects muscle tissue, that the mechanisms that build and repair external barriers are evolutionarily conserved among insects and mammals [3], [4], [5], and that our previous findings showed that *P. aeruginosa* impedes the expression of muscle cytoskeleton genes [6], prompted us to use a *Drosophila* injury and infection model as an adjunct to mouse models, to examine the pathophysiology of trauma and the relationship between injury and infection. The *Drosophila* injury and infection model has been successfully utilized to study host-*P. aeruginosa* interactions and to identify bacterial [7], [8] and host factors [6] involved in *P. aeruginosa* infection.

*P. aeruginosa* is a highly successful and virulent opportunistic human pathogen; it is the foremost agent of sepsis in severely injured patients [9]. This widespread bacterium has a diverse host range, including mammals, insects, and plants [10], [11]. Indeed, the *P. aeruginosa* PA14 clinical isolate employs a shared subset of virulence factors in insects and mice; and both hosts use conserved pathways to govern innate immunity responses to combat *P. aeruginosa* [12], [13].

Microbe recognition involves Toll/Imd signaling in flies and involves TLR4/TNFR1 in humans [12], [13], [14]. These pathways interact with the cJun-N-terminal Kinase (JNK) pathway to mediate apoptosis and cell proliferation [13]. The JNK pathway is quickly activated upon injury [15], and plays an important role in development, apoptosis, stress responses, and tissue repair in *Drosophila* and mouse [4], [5], [13], [15], [16], [17]. This pathway can be activated via the same signaling cascades that induce NF-κB mediated innate immune responses [17], [18]. Though the JNK and NF-κB signaling pathways appear to cooperate in the control of *Drosophila* immune responses [19], the precise role of JNK pathway and downstream targets in host immune responses remains unclear.

Here we uncover the role of skeletal muscle genes (SMGs) expression in hypersusceptibilty to infection that follows severe trauma and show that the presence of highly virulent *P. aeruginosa* in injured skeletal muscle tissue restricts SMG expression. Our results also demonstrate that JNK kinase Hep is involved in regulating the expression of SMGs and show that infection effects on SMG modulation are mediated locally at the site of injury and infection but not systemically. These findings may aid in developing treatments to reduce susceptibility to infection and mortality of severely injured patients.

## Results

### *P. aeruginosa* suppresses SMG regulatory network response

**Figure 1:**
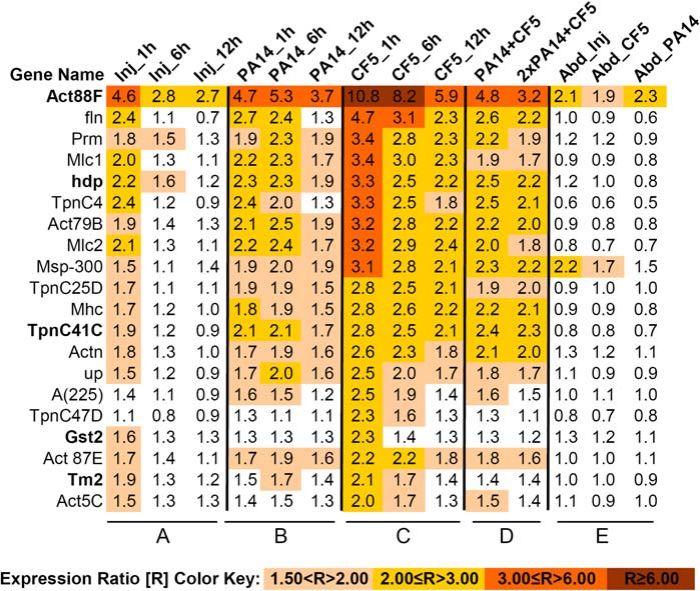
Highly virulent *P. aeruginosa* actively restricts skeletal muscle gene induction within 1 h post-inoculation. Relative expression ratio levels of 20 fly genes that encode proteins with essential functions of thorax skeletal muscle, under different conditions: 1, 6, and 12 h after thoracic injury only (A), thoracic injury and PA14 inoculation (B), or thoracic injury and CF5 inoculation (C); 1 h following thoracic injury and co-infection with 100∶100 and 200∶100 PA14:CF5 CFUs/fly (D); or following abdominal injury alone or together with inoculation with PA14 or CF5 (E). The relative expression ratio levels of selected SMGs for each condition were calculated versus naïve. The statistical evaluation of differences in expression values between PA14 and CF5 inoculated flies is presented in Table S1.

In a previous study we profiled and compared the host responses that underlie susceptible versus non-susceptible *D. melanogaster*-*P. aeruginos*a interactions using PA14 and CF5 [6], two human *P. aeruginosa* isolates that are highly virulent and non-virulent in flies, respectively [8]. Our results showed that the fly whole genome expression responses to thorax infection by these two strains were strikingly different. We identified pathogenesis- and defense-specific genes with putative roles in pathogen detection, activation of immunity signal transduction pathways, and defense; as well as non-immunity activities [6]. Part of the non-immunity related genes we identified twenty SMGs that encode proteins required for the wild-type function of thorax skeletal muscle tissue, including *Actin 88F (Act88F), Tropomyocin 2 (Tm2), held up (hdp)*, and *Glutathione S transferase 2 (Gst2)* were up-regulated 1 h post-injury relative to naïve flies (Fig. 1A). SMG up-regulation was greater in response to CF5 than PA14, especially during early (1–12 h) infection [Fig. 1B, C and Supplementary Information (SI) Table S1]. For instance, Act88F is induced 10.8-fold by CF5 and only 4.7-fold by PA14 inoculation, almost equal to the response to injury alone (Fig. 1A–C). This difference in gene expression was confirmed by the *act88F-lacZ* reporter gene [SI Fig. S1]. Such a difference in expression could be due to PA14 triggering less SMG up-regulation than CF5. Alternatively, PA14 could suppress a host pathway that signals SMG induction in response to injury and infection. SMG up-regulation at 1 h post-inoculation was lower in response to PA14 plus CF5 than CF5 alone, and similar to that triggered solely by PA14 (Fig. 1D). This suppression is likely not due to apoptosis, as PA14 does not cause noticeable apoptotic gene expression within the first 12 h of infection [6]. Also the differential SMG response is not due to growth disparity, as these strains proliferate equally in the fly thorax through 12 h post-inoculation [6]. These results suggest that PA14 actively restricts SMG induction.

### SMG function is an important component of local susceptibility to infection

**Figure 2:**
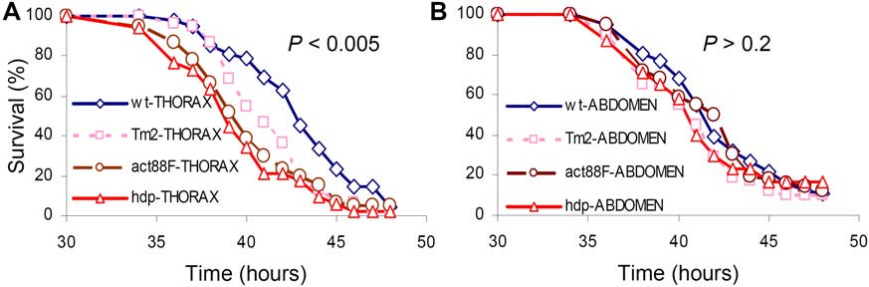
Skeletal muscle structural gene function contributes to host hypersusceptibility to infection. Survival kinetics of mutants for the flight muscle genes *act88F*, *hdp,* or *Tm2* following local PA14 infection of the thorax (A) or the abdomen (B) relative to wild-type flies. Detailed statistical evaluations of the survival kinetics are presented in Table S2.

What does decrease SMG expression mean to the fly? For instance, does it promote host susceptibility to bacterial infection, and how; and is it local or systemic? Survival kinetics of the *act88F*, *Tm2, and hdp* mutant flies, defective in muscle structure and function [20], succumbed significantly earlier to thoracic PA14 infection than wild-type flies (Fig. 2A and SI Table S2). This increased susceptibility was local and thorax specific, as the survival kinetics of abdominally injured and infected wild-type and mutant flies did not differ (Fig. 2B and Table S2). Moreover, no (or only negligible) SMG upregulation was observed with abdominal infection (Fig. 1E), as SMG expression in flies that were abdominally injured and inoculated with CF5 or PA14 was similar to that in mock-inoculated and naïve flies. These results further confirm that SMG upregulation is thorax-specific (compare Figure 1A–D to 1E), and suggest that SMGs affect local muscular susceptibility to infection.

### Wild-type expression levels of Troponin C41C and GST2 in muscle are important for susceptibility to infection at the injury site

**Figure 3:**
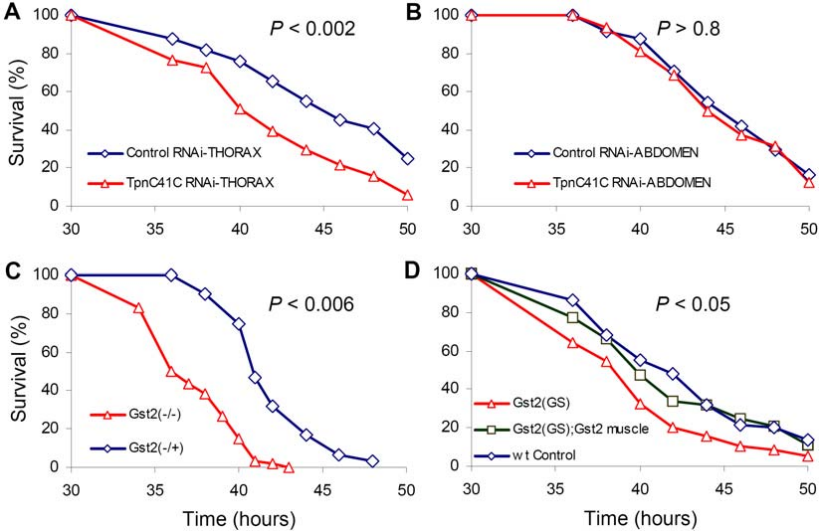
Loss of muscle specific expression of *TpnC41C* and G*st2* increases fly susceptibility to infection. Survival kinetics of flies carrying the RNAi control gene *yuri* (blue diamonds), versus muscle specific *TpnC41C* RNAi transgenic flies (red triangles), following thoracic (A) or abdominal (B) PA14 infection. (C) Homozygous flies bearing two distinct loss-of-function *Gst2^06253/04227^*alleles, presented as *Gst2(−/−)*, versus the corresponding heterozygous *Gst2^06253/+^* flies, presented as *Gst2(−/+)*. (D) Wild-type flies, versus *Gst2^GS2160^* loss of function flies, presented as Gst2(GS), and overexpression of the fly muscle specific gene *Gst2* in the *Gst2^GS2160^* loss of function background flies presented as Gst2(GS);Gst2 muscle following local PA14 infection of the thorax. Detailed statistical evaluations of the survival kinetics are presented in Table S2.

To assess the impact of SMG expression on susceptibility to infection, we knocked down expression of the fly skeletal muscle-specific *TpnC41C* gene [21] using a *TpnC41C* RNA hairpin construct. An ∼6.5-fold reduction in *TpnC41C* expression was confirmed by quantitative RT-PCR (SI Fig. S2A). *TpnC41C* knockdown expression rendered flies more susceptible (Table S2) to thoracic (Fig. 3A), but not abdominal (Fig. 3B), infection.

The expression of another SMG gene, *Glutathione S-Transferase 2 (Gst2)*, which belongs to a family of detoxification genes [22], is also limited following PA14 infection (Fig. 1). Gst2 is highly abundant in the flight muscle and nervous system of *Drosophila* [23], [24], and is physically linked to muscle fibers via its interaction with Troponin H [24]. *Gst2* expression increases in response to injury, and is further stimulated by CF5, but not PA14, inoculation (Fig. 1). Homozygous *Gst2* mutant flies, *Gst2^06253/04227^*, are more susceptible to PA14 pathogenesis than heterozygous flies, *Gst2^06253/+^* or *Gst2^+/04227^* (Fig. 3C and Table S2). RT-PCR showed that *Gst2^06253/04227^* flies have ∼2-fold lower *Gst2*, expression than heterozygous flies despite equal *Act88F* RNA levels (SI Fig. S2B). Furthermore, flies homozygous for a weak hypomorphic allele of *Gst2* (*Gst2^GS2160^*) exhibited ∼40% lower RNA levels (SI Fig. 2C) and died faster than wild-type controls (Fig. 3D and Table S2).

To test whether Gst2 expression in muscle alters susceptibility to *P. aeruginosa* infection, we directed the expression of Gst2 using a muscle-specific gene expression driver (dMef2-GAL4). Gst2 expression was reestablished specifically in the muscle of Gst2 mutants (muscle-specific Gst2 “rescue”). Muscle-specific overexpression of *Gst2* in the weak hypomorphic background (*Gst2^GS2160^;dMef2-GAL4/+)* induced ∼10-fold increases in *Gst2* RNA levels (SI Fig. S2C), and produced post-PA14 infection mortality kinetics similar to that of wild-type flies (Fig. 3D and Table S2). These data demonstrate that Gst2 in muscle affects susceptibility to infection and may constitute a functional component of the host “defense” response to *P. aeruginosa* infection that it is independent of antimicrobial peptides (AMPs), well known host defense effectors. Indeed, expression of *Diptericin* (*Dipt*), which is considered the most representative AMP response against Gram-negative bacteria, was not altered in any of the transgenic flies tested (SI Fig. S2A–C).

### The JNK pathway controls the early induction of skeletal muscle genes via Hep

Although the JNK pathway is induced in various tissues, it has been specifically linked to muscle gene expression in *Drosophila* embryos and the muscle gene *flightin* is induced by JNK upon bacterial inoculation [17], [25]. To assess if the JNK pathway regulates SMG expression, we used *hep^1^* flies, which carry a hypomorphic mutation of the JNK kinase gene *hep*, resulting in reduced JNK pathway activity [26]. Null mutations in JNK pathway components could not be used as they are typically embryonic lethal. We subjected *hep^1^* flies to injury and CF5 strain inoculation in the thorax and compared their expression profiles to those observed with wild-type flies. Unlike our observations in wild-type flies, SMG induction was nearly absent in *hep^1^* flies following injury and CF5 inoculation (SI Fig. S3). These findings demonstrate that Hep regulates the expression of 20 SMGs and affects susceptibility to *P. aeruginosa* infection at the site of injury.

### Hep restricts bacterial growth at the injury site and reduces mortality

**Figure 4:**
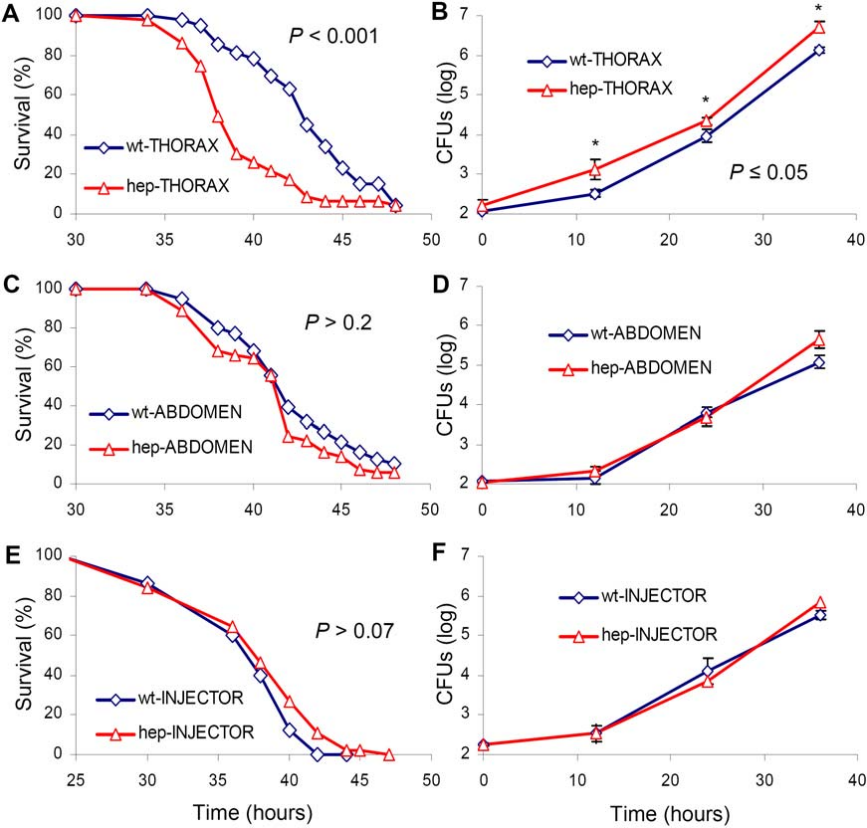
*hep* mediates increased resistance to thoracic, but not abdominal or systemic, infection. Survival kinetics of (A, C, E) and bacterial proliferation per fly in (B, D, F) wild-type and *hep^1^* flies, following local PA14 infection of the thorax (A, B), or the abdomen (C, D), or systemic injector-pumping PA14 infection (E, F). Error bars indicate Standard Deviation of the mean (C, D, F) and * indicate t-test *P*-values of ≤0.05 (B). The difference in mortality kinetics between wild type and mutant flies is statistically significant. Detailed statistical evaluations of the survival kinetics are presented in Table S2.

We compared the survival kinetics of mutant and wild-type flies after local thoracic, abdominal or systemic PA14 infection (Fig. 4A,C,E), accomplished by needle-pricking (local) or injector-mediated pumping (systemic). The *hep^1^* flies died significantly earlier than wild-type flies after thoracic (Fig. 4A), but not after abdominal (Fig. 4C) or injector-mediated infection (Fig. 4E). Furthermore, relative to similarly treated wild-type flies, greater bacterial growth was observed in the thorax-inoculated *hep^1^* flies (Fig. 4B), but not abdomen-inoculated (Fig. 4D) or systemically-infected (Fig. 4F) *hep^1^* flies.

To further assess the importance of *hep^1^* in the susceptibility to infection at the site of injury, *hep^1^* flies infected in the thorax were dissected and colony forming units (CFUs) were assessed in thorax and abdomen over time. Higher CFUs were observed in the thorax, but not the abdomen, of *hep^1^* flies (SI Fig. S4). Furthermore, Hep overexpression via the muscle-specific driver dMef2-GAL4 (Mef-G4) [27] rendered flies more resistant to infection than isogenic wild-type flies (SI Fig. S5A). As expected, Hep overexpression increased *act88F* expression within 1 h of infection (SI Fig. S5B). In addition, flies expressing a dominant negative version of the gene, DNJNK (DNBsk), specifically in the muscle are more susceptible to thoracic (SI Fig. S5C), but not systemic (SI Fig. S5D) infection. These results indicate that Hep and JNK function to protect against *P. aeruginosa* infection likely via increased expression of genes that mediate local tissue reconstruction following injury and infection.

To determine whether maximal Hep activity is merely important for muscle response to injury and infection, rather than muscle development and structure, we assessed the effect of *hep^1^* mutation on muscle structure and function. Fly muscle ultrastructure viewed by electron microscopy (SI Fig. S6), flight ability and antigravity walking of *hep^1^* flies were comparable to that of wild-type flies. Therefore, modulation of Hep activity in *hep^1^* flies does not perturb muscle development or structure, but rather affects expression of SMGs, whose absence increases susceptibility to *P. aeruginosa* infection.

### JNK activation functions in the mammalian local defense response to *P. aeruginosa* infection in muscle

**Figure 5:**
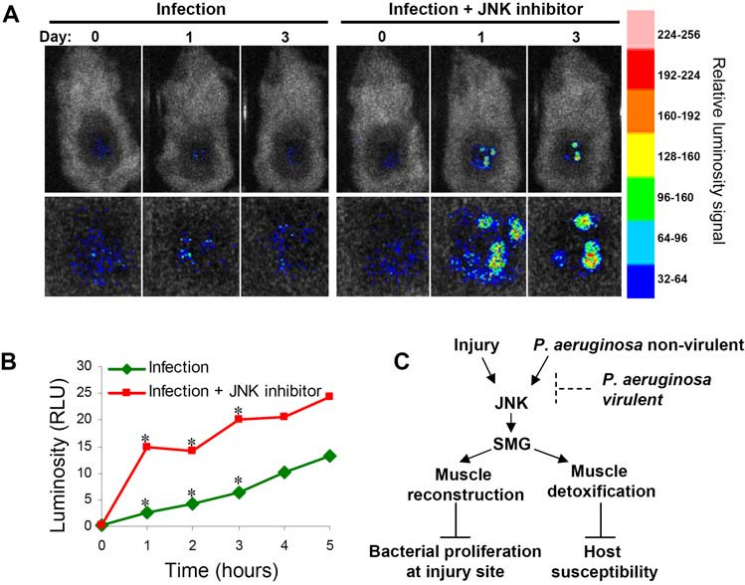
JNK inhibition increases *P. aeruginosa* proliferation in mouse muscle. (A) “Infection” and “Infection + JNK inhibitor” mice were respectively injected with empty micelles and micelles filled with the JNK inhibitor SP600125, and then inoculated with bioluminescent *P. aeruginosa* cells. The inhibitor did not perturb bacterial growth *in vitro*. Luminescence was assessed 0, 1, and 3 d post-treatment. The luminescence signal is magnified 3X in the lower panels. (B) Average luminescence from eight control versus eight JNK inhibitor mice. *P≤0.05 for days 1, 2, and 3 based on t- and Wilcoxon tests. RLU, relative luminescence units. (C) Diagram depicting the proposed role of JNK pathway in host defense and the ability of *P. aeruginosa* to affect SMGs expression.

To determine whether the role of the JNK pathway in susceptibility to *P. aeruginosa* infection is functionally conserved in mammals, *P. aeruginosa* proliferation was assessed over time in the mouse open wound infection model [28]. Injection of the specific JNK inhibitor SP600125 [29] into the spinalis muscle increased luminescence over time and was significantly higher in the JNK inhibitor-exposed mice, which emitted a more intense signal on days 1–3 post-infection than infected control mice (Fig. 5). This effect, which was produced by a single injection of the inhibitor into muscle immediately prior to inoculation, but not after systemic intravenous injection (not shown), showed that JNK functions in the mouse to mediate the local defense response to *P. aeruginosa* in injured muscle.

## Discussion

Here we identified and demonstrated that a set of SMGs constitutes host genetic components that contribute to the local hyper-susceptibility to *P. aeruginosa* infection in injured tissue, providing novel insights into the pathophysiology of trauma and the relationship between injury and infection. Our results showed that highly virulent *P. aeruginosa* cells actively impeded upregulation of protective SMG responses at the site of injury, while non-virulent *P. aeruginosa* cells promoted SMG upregulation. Furthermore, we found that SMG activation was tightly controlled by the JNK kinase Hep, whose activation at the injury site restricted bacterial growth and delayed mortality, linking JNK signaling to the hyper-susceptibility to infection in trauma. Functional expression studies that assess the role of specific SMGs in restricting infection at the site of injury, demonstrated that wild-type expression of the skeletal muscle structural genes *TroponinC41C* and *Gst2* play an early role in restricting infection at the injury site.

Local and systemic SMG responses could be distinguished in our experimental model. SMG expression is induced by thoracic injury alone, a fact consistent with SMG involvement in tissue repair. However, SMG expression is prolonged when bacteria are present in the injury, with bacterially-mediated up-regulation being significantly higher in response to non-virulent CF5 versus virulent PA14 cells, especially during early infection. Although PA14 infection produces additional tissue damage, and presumably a greater need for muscle restoration, SMG expression was not, or only slightly, upregulated beyond that seen with injury. Hence PA14 cells should have access to nutrients released in the microenvironment of the wounded muscle, promoting more aggressive proliferation. Indeed, we showed that thoracic injury and PA14 inoculation promotes high CFU titers locally, and have observed muscle fiber disruption[8] within 24 h of thoracic injury and infection. Such damage is absent in CF5 inoculated flies (data not shown).

As in flies, involvement of muscle tissue in response to external injury is commonly observed in mammals. Following skin damage, underlying muscle may provide a physical barrier to pathogen invasion. In this regard, SMG expression could counteract muscle tissue decay by replenishing muscle structure components. Another aspect of muscle “resistance” to bacterial proliferation could be its detoxification properties. Muscle gene expression includes the upregulation of *Gst2*, a gene with detoxification properties [30]. By scavenging lipid peroxidation radicals [30], Gst2 can potentially increase the tolerance of infected tissue to cytotoxicity and cell death. Therefore, muscle structure support mediated by structural component restoration and possible tissue detoxification via Gst2 could act in concert to keep muscle destruction in check and limit bacterial proliferation during infection (Figure 5C).

The induction of SMG expression in response to infection is coupled to antimicrobial peptide gene expression through a common upstream signaling pathway [17], [18]. Our work shows that SMG induction acts as a homeostatic mechanism to support host defenses. This response can be a complementary defense mechanism to the increased systemic antimicrobial peptide production following injury and infection. Similarly, findings of a functional genomics analysis of mosquito responses to parasite invasion, suggested that cytoskeletal gene induction of the local epithelia constitute, together with the systemic humoral immunity, an integrated defense against infection [31]. Notably, the JNK-dependent SMG-mediated “defense” response against *P. aeruginosa* relies on small differences in gene expression levels. We propose that it is the collective response of many muscle genes that orchestrates muscle tissue restoration. Moreover, because the number of muscle cells injured and infected at the early time points are presumably a small percentage of the total, the expression profile of the whole fly reflects an underestimation of gene expression in the immediately affected muscle cells. Furthermore, small differences in gene expression can determine risk for specific human diseases [32]. Since the JNK signaling cascade is important for multiple-defense related loci, the JNK-pathway network may also include clinically significant muscle gene loci. Downregulation of SMG expression in *hep^1^* flies could indicate a general role for the JNK pathway in muscle cytoskeleton homeostasis, similar to the role of JNK in neuronal cytoskeleton maintenance [33].

Our results also showed that JNK-mediated responses also function in mammals to restrict *P. aeruginosa* infections. Accordingly, local pharmacological potentiation of this pathway could provide a novel anti-infective approach to prevent deleterious *P. aeruginosa* wound infections that can lead to systemic sepsis in mammals. The development of approaches that modulate the JNK host response or interfere with its pathogen suppression could provide new treatments to limit infections in susceptible patients.

## Materials and Methods

### Bacteria

The *P. aeruginosa* human isolate PA14 was described previously [8], [11]. S. Lory kindly provided the human CF5 isolate [34]. *P. aeruginosa* ATCC 19660 (Xen5), which carries the bioluminescent lux operon as a chromosomal insertion, was a gift from Xenogen, Inc. (Alameida, CA). Xen5 is equally highly virulent in flies and mice as PA14 (data not shown). None of the *P. aeruginosa* strains exhibited *in vitro* growth defects.

### Flies

Canton-S (Bloomington Stock Center, BSC#1) was used as the wild-type control strain. Unless otherwise indicated, mutant strains were homozygotes. *hep^1^* is a hypomorphic mutation, and *act88F^KM129^* is a 3′ deletion (gifts from M. Miura and Y. Hotta, respectively); *Gst2^04227^* and *Gst2^06253^* (BSC#11367 and 11480) are P-element promoter insertions; *Tm2^3^* (BSC#4053) is a hypomorphic mutation; *hdp^2^* is a coding region point mutation (gift from J.C. Sparrow); and the Gst2 overexpression strain (*Gst2^GS2160^*; gift from T. Aigaki) is a GAL4-inducible gene trap insertion in the Gst2 locus causing reduced gene expression when not induced. To induce Gst2 in a Gst2 hypomorphic background, *Gst2^GS2160^* homozygous flies carrying one copy of the dMef2-Gal4 driver were used (gift from N. Perrimon). Act88F-lacZ flies [35] were used as heterozygotes (gift from K. VijayRaghavan). UAS-hep (BSC#9308) and UAS-DNJNK (BSC#6409) flies were crossed with dMef2-GAL4 for *hep* overexpression respectively. For survival studies, *w^+^* marked *Gst2^GS2160^*, UAS-*hep* and UAS-DNJNK flies were backcrossed 6 times to *w^1118^* flies to create an isogenic background. For TpnC41C RNAi, we crossed the dMef2-GAL4 driver with the UAS-TpnC41C RNAi transgene construct 2981R-2, and with the UAS-yuri RNAi transgene construct 4584R-3 as a control (National Institute of Genetics, Japan; http://shigen.lab.nig.ac.jp/fly/nigfly/).

### Infection assessment assays

Fly maintenance and infection assays were conducted as described previously [8], [36]. For the survival assays, ≥50 flies were infected with a bacterial suspension (grown in LB to OD_600_ 3) and maintained at 21°C. Each experiment was performed at least in triplicate with qualitatively similar results. Bacterial inoculations were accomplished via pricking the middle dorsolateral thorax [8] or the anterior dorsolateral abdomen of the fly with a needle previously dipped into the bacterial suspension of 5×10^7 ^or injecting 13.8 nl of the bacterial of the 10^7 ^solution into the middle dorsolateral thorax (Nanoject II, Drummond). The number of inoculated cells following pricking was ∼100 per fly. Distribution of bacterial cells spread through the body of the fly was assessed by separating the fly thorax from the abdomen with fine forceps minutes after bacterial inoculation. Fly thorax and abdomen separately grinded and plated on LB agar plates at the appropriate dilutions for colony forming units (CFUs) and Standard Deviation (SD) assessment. Thoracic needle-pricking delivered 94%±3% CFUs in the thorax (site of inoculation) and 6±3% in the abdomen (remote site). While, abdominal needle-pricking delivered 51±10% CFUs in the thorax (remote site) and 49±10% in the abdomen (site of inoculation). Finally, thoracic injector-pumping delivered in 64±5% CFUs in the thorax (site of inoculation) and 36±5% in the abdomen (remote site). For CFU assessment, 3 groups of 4 flies each were used for each time point and condition. Mortality due to injury was always less than 10% and occurred within the fist 24 h post-injury. The ∼5% of flies that died within 24 h post-injury and -inoculation were excluded from the study.

The mouse open wound infection model [28] was used to determine *P. aeruginosa* (Xen5) cell proliferation over time, via *in vivo* serial imaging of bacterial bioluminescence in live mice using a Hamamatsu low light camera. Following injury, a single 700 mg dose of the JNK pathway inhibitor pyrazolanthrone [SP600125, A.G. Scientific [29]] was administered. This dose was previously shown to specifically inhibit mouse JNK [37]. The drug was packaged in micelles and injected into the spinalis muscle just below the panniculus carnosus collagen layer. The exposed collagen tissue was then inoculated with 2×10^6^ Xen5 cells (grown in LB to OD_600_ 3). Eight mice were injected with the drug and 8 control mice were injected with empty micelles prior to their infection. Wound bioluminescence was determined using Argus III software by multiplying the mean intensity of the area of luminescence by the number of pixels.

### Micelle preparation

Pyrazolanthrone (5 mg/ml) was dissolved in methanol with stirring at 45°C. Polyoxyethylenglyceroltriricinoleate (Cremophor EL, BASF AG) was added (32∶1 w/w; 2.9∶1 mol/mol), and the methanol was evaporated under a nitrogen stream at 50°C, and then at 80°C with weight monitoring. Micelles were subsequently formed by the addition of 0.9% NaCl buffered with 50 mM HEPES, pH 7, at 50°C. This solution was filtered at 80°C through a 100 nm Nucleopore membrane. The pyrazolanthrone concentration was determined photometrically by measuring its absorption at 396 nm (1∶100 dilution in methanol).

### Statistical analysis

Fly survival kinetics were analyzed using R software (www.r-project.org/). Mutant survival curves were analyzed using the log-rank test (Mantel-Haenszel) of the Kaplan-Meier estimate of survival [38], and the Cox proportional hazards regression model [39], to test the null hypothesis that the mutant and wild-type survival kinetics were equivalent. Bioluminescence differences were determined using t- and Wilcoxon rank sum tests [40].

### Microarray analysis

Fifty flies were snap frozen immediately after treatment and grinded to extract RNA using RNAeasy kit (Qiagen). Affymetrix GeneChip® Drosophila Genome Arrays were used. Labeling, hybridization and scanning were done as previously described [6]. All conditions were performed in triplicate and produced qualitatively similar results. The raw scanned image files were processed by using data normalization, quality assurance and control, filtering, and clustering. Stable invariant-set normalization and perfect match model-based expression values were generated by using the DCHIP program (www.biostat.harvard.edu/complab/dchip). All arrays passed quality control by DCHIP. A probe set was eliminated if it showed small variation (SD/mean <0.2) across samples (after pooling replicate arrays) or an overall signal intensity at or below background [4×rawQ (a measure of noise value, representing the degree of pixel to pixel variation of probe cells on a GeneChip array)]. A dual filter was applied to identify differentially expressed genes in flies infected with PA14 or CF5 by the following criteria: (i) each gene had at least a 1.5-fold change in its expression value between either PA14 and injury or CF5 and injury for at least one time point; and (ii) the gene expression value had at least a 1.5-fold change between PA14 and CF5 for at least one time point. Each raw scanned image was normalized and subjected to quality assurance and control. Subsequently we selected 20 muscle cytoskeletal genes (according to the FLYBASE gene ontology database at http://flybase.bio.indiana.edu/) showing at least 2-fold higher expression in CF5 *vs.* PA14 thoracic inoculation. Statistical evaluation using t-test assesses the differences in between PA14 and CF5 in thoracic and abdominal infection and P-values are presented in Table S1. CEL files are deposited to MIAMExpress (accession number E-MEXP-1287).

### Scanning electron microscopy

Flies were fixed in 4.0% paraformaldehyde, 3.5% glutaraldehyde (Electron Microscopy Sciences, Hatfield, PA) with 1.0% w/v tannic acid in 0.1 M sodium cacodylate buffer, pH 7.4 overnight at 4°C, rinsed in buffer, post-fixed in 1% osmium tetroxide in cacodylate buffer for 1 h at room temperature (RT), rinsed in buffer, then in distilled water and stained, *en bloc*, in an aqueous solution of 2.0% uranyl acetate for 1 h at RT. Subsequently samples were rinsed in distilled water and dehydrated through a graded series of ethanol to 100%, and infiltrated with Epon resin (Ted Pella, Redding, CA) in a 1∶1 solution of Epon:ethanol. The following day they were placed in fresh Epon for several hours and then embedded in Epon overnight at 60°C. Thin sections were cut on a Reichert Ultracut E ultramicrotome, collected on formvar-coated slot grids, stained with uranyl acetate and lead citrate and examined in a JEOL JEM 1011 transmission electron microscope at 80 kV. Images were collected using an AMT (Advanced Microscopy Techniques, Danvers, MA) digital imaging system.

### Quantitative RT-PCR

Quantitative RT-PCR assays were preformed according to the manufacturer's instructions. Briefly, for each experiment, total RNA was extracted from 5–10 male flies with Trizol® Reagent (Invitrogen) and subsequently purified with RNAeasy Plus and column DNase I treatment (Qiagen). One microgram of total RNA was treated with reverse transcriptase (Superscript II; Invitrogen) for 1 h. Reactions were prepared using an SYBR Green Kit (Applied Biosystems) and run in an ABI 9700HT RT-PCR machine. Melting curves were used to exclude samples with genomic DNA and to ensure single product amplification. CG6213 was used as internal normalization control. We used the following pairs of primers: *Gst2* 5′-AACGATGGTCACCTGGCTCT-3′ and 5′-GGTGATGCCTGCGAAGTAGAC-3′; *Act88F* 5′-CGGTATCCTGACGCTGAAGT-3′ and 5′-CTTCTCCATGTCGTCCCAGT-3′; *Diptericin* 5′-TGTGAATCTGCAGCCTGAAC-3′ and 5′-GCACATCAAAATTGGGAGCATA-3′; *GC6213* 5′-CCAAACTGTGCGCTGTAAATTC-3′ and 5′-TTATGGATTAGAGTTGAACGTGGA-3′; *TpnC41C* 5′-TTATCGCTGAAGTCGATGAGGA-3′ and 5′-GCCAGGGTGGTAAATTCTTCAA-3′. We used the 1/2^C^ formula, where C is the estimated threshold cycle value, to calculate relative expression levels. Values for different genes were adjusted to similar scale for presentation. All experiments were performed in triplicate.

## Supporting Information

Figure S1 Higher induction of act88F-lacZ in transgenic flies inoculated with the CF5 versus PA14 strain. Forty female flies heterozygous for the act88F-lacZ transgene were either left untreated, or subjected to thoracic needle-mediated bacterial inoculation with PA14 or CF5 cells. Flies were ground up in PBS and LacZ levels were assesses via ONPG/LacZ liquid assays and presented as Miller units. Experiments were done in triplicate and the difference between PA14 and CF5 inoculated flies was statistically significant (*P = 0.01; two tailed t-test).
(0.18 MB PDF)

Figure S2 Gst2 and TpnC41C RNA levels are reduced in Gst2 mutant and TpnC41C RNAi flies, respectively. (A) Relative RNA levels of TpnC41C, Gst2 and Dipt in flies of the UAS-yuriRNAi/+;dMef2-GAL4/+ and UAS-TpnC41CRNAi/+;dMef2-GAL4/+ genotypes, presented as Control RNAi and TpnC41C RNAi respectively. (B,C) Relative RNA levels of Gst2, act88F and Dipt in flies of the following genotypes: Gst206253/04227allele, presented as Gst2(−/−) and Gst206253/+, flies presented as Gst2(−/+). (B) and w1118, presented as wt Control, Gst2GS2160, presented as Gst2(GS) and Gst2GS2160;dMef2-GAL4/+ (overexpression of the fly muscle specific gene Gst2 in the Gst2GS2160 loss of function background flies), presented as Gst2(GS);Gst2 muscle (C). TpnC41C levels in the TpnC41C RNAi group differed from that in the Control RNAi group in A (*P = 0.02; two tailed t-test) and Gst2 levels in Gst206253/04227 differed from that of Gst206253/+in B and Gst2GS2160 differed from that of w1118 and Gst2GS2160;dMef2-GAL4/+ in C (*P<0.01; two tailed t-test).
(0.18 MB PDF)

Figure S3 Hep mediates collective expression of SMGs. Expression ratio of SMGs in wild-type and hep1 flies in thoracic injured, or injured and inoculated in the thorax with CF5 strain conditions 1 h post-treatment. The relative expression ratio levels of SMGs for each condition were calculated versus naïve for each genotype.
(0.17 MB PDF)

Figure S4 hep1 mutation allows higher bacterial proliferation in the thorax but not the abdomen. Comparison of CFUs over time of wild-type and hep1 fly thoraces (A) and abdomens (B) collected from the same flies that had been inoculated in the thorax. Error bars indicate Standard Deviation of the mean.
(0.17 MB PDF)

Figure S5 Muscle specific hep overexpression increases fly survival of PA14 infection. (A) Survival kinetics of wild-type flies with (Mef-G4/U-hep), or without (Mef-G4/+), specific overexpression of Hep in skeletal muscle following thoracic PA14 infection. (B) Hep-overexpressing flies had augmented act88F (*P = 0.007; two tailed t-test), but not Dipt, transcription 1 h post-infection. Survival kinetics of flies overexpressing the dominant negative form of JNK (DNJNK) in the muscle following thoracic (C) or systemic, injector-mediated (D) infection. Kaplan-Meier P-values of the survival kinetics are presented on the graphs. Error bars indicate Standard Deviation of the mean.
(0.17 MB PDF)

Figure S6 hep1 mutation does not disrupt muscle structure. Wild-type (A) and hep1 (B) fly muscle structure, viewed by transmission electron microscopy.
(0.17 MB PDF)

Table S1 Student t-test P-values of the 20 SMGs assessing the difference in expression in PA14 vs CF5 needle-pricking inoculated flies (1, 6 and 12 hours post- thoracic or abdominal treatment).
(0.02 MB PDF)

Table S2 Statistical analysis of fly survival post *P. aeruginosa* infection using the Kaplan-Meier (1) and Cox (2) models. Comparison of the survival curve P values as derived from the Mantel-Haenszel test using the Kaplan-Meier estimate of survival (1); and the likelihood ratio test using the Cox proportional hazards regression model (2). P values of less than 0.05, using both models, are considered to indicate significant differences. (1) Kaplan, E., and P. Meier. 1958. Nonparametric estimation from incomplete observations. J. Am. Stat. Assoc. 53:457–481, 562–563. (2) Cox, D. R. 1972. Regression models and life tables. J. Royal Stat. Soc. Ser. B 34:187–220.
(0.44 MB PDF)
