# Supplementary figures and images for: Involvement of Skeletal Muscle Gene Regulatory Network in Susceptibility to Wound Infection Following Trauma
Source: PLoS One. 2007 Dec 26;2(12):e1356. doi: 10.1371/journal.pone.0001356 (PMC2131783; doi:10.1371/journal.pone.0001356)

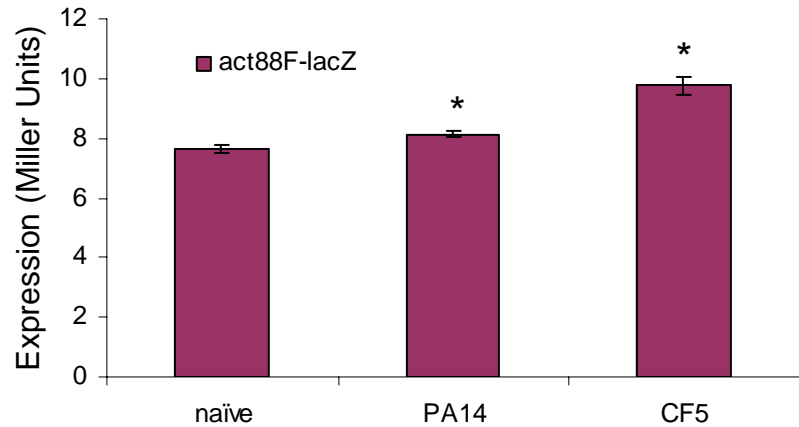

Supplement: Figure S1 — Higher induction of act88F-lacZ in transgenic flies inoculated with the CF5 versus PA14 strain. Forty female flies heterozygous for the act88F-lacZ transgene were either left untreated, or subjected to thoracic needle-mediated bacterial inoculation with PA14 or CF5 cells. Flies were ground up in PBS and LacZ levels were assesses via ONPG/LacZ liquid assays and presented as Miller units. Experiments were done in triplicate and the difference between PA14 and CF5 inoculated flies was statistically significant (*P = 0.01; two tailed t-test). (0.18 MB PDF) [file pone.0001356.s001.pdf]

**A**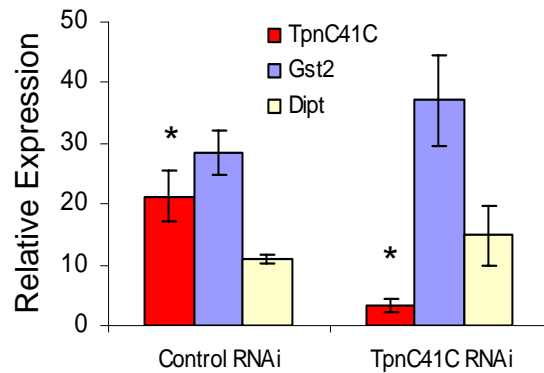**B**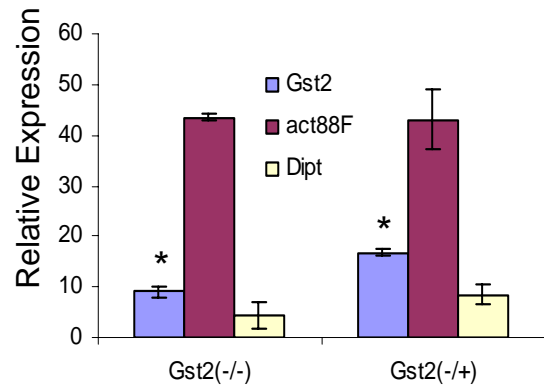**C**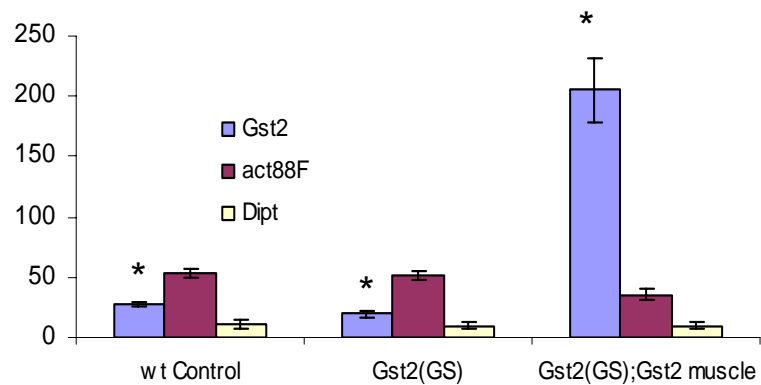

Supplement: Figure S2 — Gst2 and TpnC41C RNA levels are reduced in Gst2 mutant and TpnC41C RNAi flies, respectively. (A) Relative RNA levels of TpnC41C, Gst2 and Dipt in flies of the UAS-yuriRNAi/+;dMef2-GAL4/+ and UAS-TpnC41CRNAi/+;dMef2-GAL4/+ genotypes, presented as Control RNAi and TpnC41C RNAi respectively. (B,C) Relative RNA levels of Gst2, act88F and Dipt in flies of the following genotypes: Gst206253/04227allele, presented as Gst2(−/−) and Gst206253/+, flies presented as Gst2(−/+). (B) and w1118, presented as wt Control, Gst2GS2160, presented as Gst2(GS) and Gst2GS2160;dMef2-GAL4/+ (overexpression of the fly muscle specific gene Gst2 in the Gst2GS2160 loss of function background flies), presented as Gst2(GS);Gst2 muscle (C). TpnC41C levels in the TpnC41C RNAi group differed from that in the Control RNAi group in A (*P = 0.02; two tailed t-test) and Gst2 levels in Gst206253/04227 differed from that of Gst206253/+in B and Gst2GS2160 differed from that of w1118 and Gst2GS2160;dMef2-GAL4/+ in C (*P<0.01; two tailed t-test). (0.18 MB PDF) [file pone.0001356.s002.pdf]

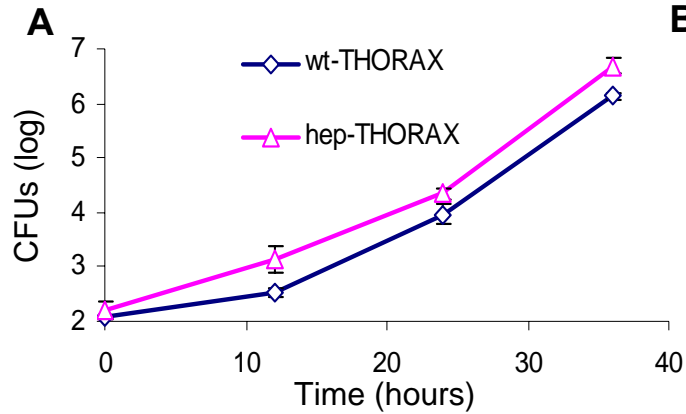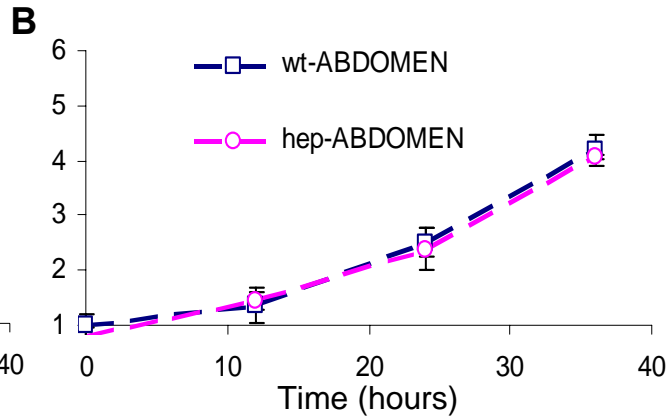

Supplement: Figure S4 — hep1 mutation allows higher bacterial proliferation in the thorax but not the abdomen. Comparison of CFUs over time of wild-type and hep1 fly thoraces (A) and abdomens (B) collected from the same flies that had been inoculated in the thorax. Error bars indicate Standard Deviation of the mean. (0.17 MB PDF) [file pone.0001356.s004.pdf]

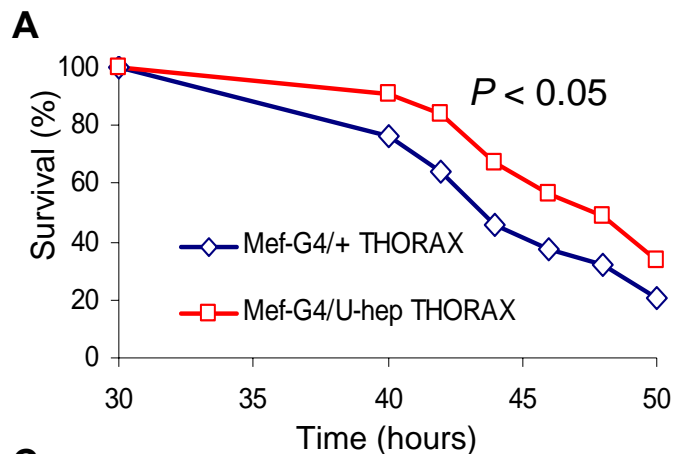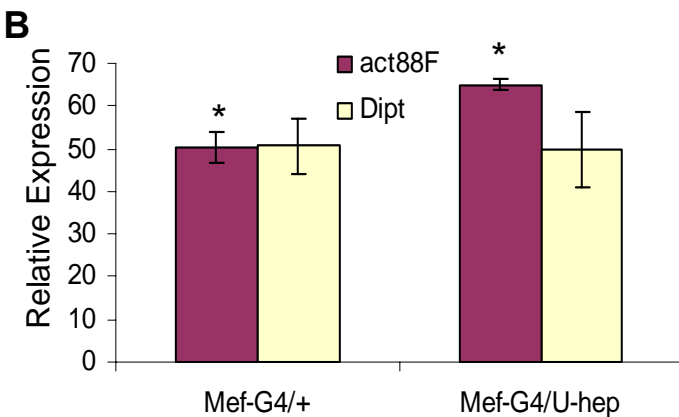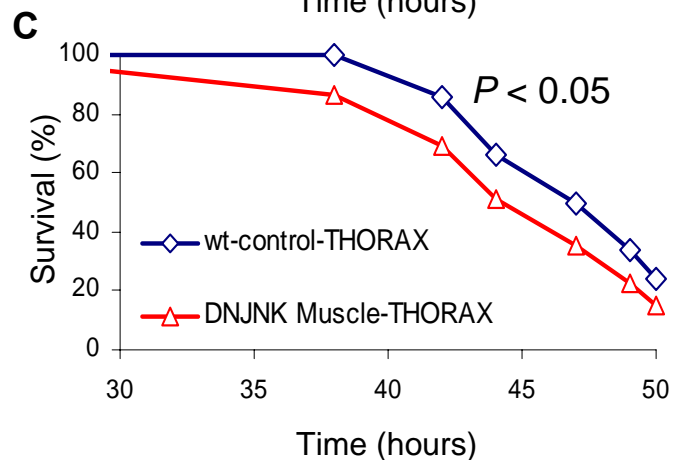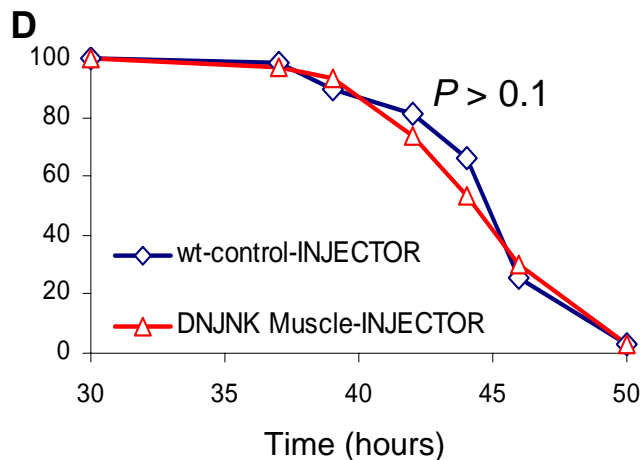

Supplement: Figure S5 — Muscle specific hep overexpression increases fly survival of PA14 infection. (A) Survival kinetics of wild-type flies with (Mef-G4/U-hep), or without (Mef-G4/+), specific overexpression of Hep in skeletal muscle following thoracic PA14 infection. (B) Hep-overexpressing flies had augmented act88F (*P = 0.007; two tailed t-test), but not Dipt, transcription 1 h post-infection. Survival kinetics of flies overexpressing the dominant negative form of JNK (DNJNK) in the muscle following thoracic (C) or systemic, injector-mediated (D) infection. Kaplan-Meier P-values of the survival kinetics are presented on the graphs. Error bars indicate Standard Deviation of the mean. (0.17 MB PDF) [file pone.0001356.s005.pdf]

## Wild type muscle EM

A

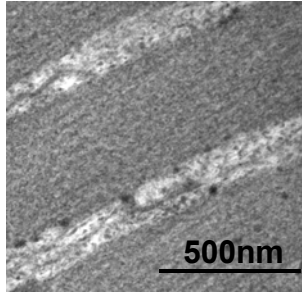

## *hep<sup>1</sup>* muscle EM

B

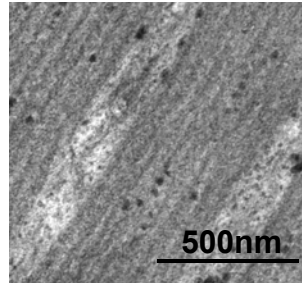

Supplement: Figure S6 — hep1 mutation does not disrupt muscle structure. Wild-type (A) and hep1 (B) fly muscle structure, viewed by transmission electron microscopy. (0.17 MB PDF) [file pone.0001356.s006.pdf]
