# Supplementary material for: Involvement of Skeletal Muscle Gene Regulatory Network in Susceptibility to Wound Infection Following Trauma
Source: PLoS One. 2007 Dec 26;2(12):e1356. doi: 10.1371/journal.pone.0001356 (PMC2131783; doi:10.1371/journal.pone.0001356)
Supplement: Figure S3 — Hep mediates collective expression of SMGs. Expression ratio of SMGs in wild-type and hep1 flies in thoracic injured, or injured and inoculated in the thorax with CF5 strain conditions 1 h post-treatment. The relative expression ratio levels of SMGs for each condition were calculated versus naïve for each genotype. (0.17 MB PDF) [file pone.0001356.s003.pdf]

Gene Name	Inj_1h/naïve	CF5_1h/naïve	hep_Inj/hep_naïve	hep_CF5/hep_naïve
**Act88F**	4.6	10.8	1.2	1.1
fln	2.4	4.7	1.2	1.0
Prm	1.8	3.4	1.2	1.1
Mlc1	2.0	3.4	1.1	1.1
**hdp**	2.2	3.3	0.9	1.2
TpnC4	2.4	3.3	1.2	1.0
Act79B	1.9	3.2	1.0	1.2
Mlc2	2.1	3.2	1.1	1.2
Msp-300	1.5	3.1	1.2	0.9
TpnC25D	1.7	2.8	1.0	1.0
Mhc	1.7	2.8	1.1	1.0
**TpnC41C**	1.9	2.8	1.0	1.0
Actn	1.8	2.6	0.9	0.8
up	1.5	2.5	1.2	1.2
A(225)	1.4	2.5	1.0	0.9
TpnC47D	1.1	2.3	0.9	0.8
**Gst2**	1.6	2.3	1.0	1.1
Act 87E	1.7	2.2	1.1	1.2
**Tm2**	1.9	2.1	0.9	1.2
Act5C	1.5	2.0	1.0	1.1

Expression Ratio [R] Color Key:

1.50<R>2.00	2.00≤R>3.00	3.00≤R>6.00	R≥6.00
