# Supplementary material for: Involvement of Skeletal Muscle Gene Regulatory Network in Susceptibility to Wound Infection Following Trauma
Source: PLoS One. 2007 Dec 26;2(12):e1356. doi: 10.1371/journal.pone.0001356 (PMC2131783; doi:10.1371/journal.pone.0001356)
Supplement: Table S1 — Student t-test P-values of the 20 SMGs assessing the difference in expression in PA14 vs CF5 needle-pricking inoculated flies (1, 6 and 12 hours post- thoracic or abdominal treatment). (0.02 MB PDF) [file pone.0001356.s007.pdf]

Gene Name	PA14 vs CF5 1h	PA14 vs CF5 6h	PA14 vs CF5 12h
Act88F	0.000	0.021	0.029
fln	0.049	0.126	0.162
Prm	0.042	0.173	0.041
Mlc1	0.060	0.171	0.058
wupA	0.127	0.357	0.304
TpnC4	0.164	0.200	0.274
Act79B	0.024	0.041	0.009
Mlc2	0.112	0.163	0.053
Msp-300	0.100	0.265	0.426
TpnC25D	0.072	0.137	0.278
Mhc	0.048	0.099	0.017
TpnC41C	0.010	0.041	0.254
Actn	0.063	0.015	0.282
up	0.348	0.418	0.460
Strn-Mlck	0.397	0.465	0.112
TpnC47D	0.107	0.194	0.301
GstS1	0.062	0.471	0.455
Act87E	0.076	0.096	0.089
Tm2	0.017	0.309	0.323
Act5C	0.085	0.112	0.197
	P-value	*P-value*	*P-value*

Statistically signifficant differences in expression are highlighted in yellow

Gene Name	PA14 vs CF5 ABDOMEN 1h
Act88F	0.216
fln	0.399
Prm	0.235
Mlc1	0.311
wupA	0.296
TpnC4	0.198
Act79B	0.405
Mlc2	0.451
Msp-300	0.229
TpnC25D	0.394
Mhc	0.202
TpnC41C	0.248
Actn	0.427
up	0.490
Strn-Mlck	0.380
TpnC47D	0.460
GstS1	0.081
Act87E	0.192
Tm2	0.254
Act5C	0.205
	P-value
