# Supplementary material for: Involvement of Skeletal Muscle Gene Regulatory Network in Susceptibility to Wound Infection Following Trauma
Source: PLoS One. 2007 Dec 26;2(12):e1356. doi: 10.1371/journal.pone.0001356 (PMC2131783; doi:10.1371/journal.pone.0001356)
Supplement: Table S2 — Statistical analysis of fly survival post P. aeruginosa infection using the Kaplan-Meier (1) and Cox (2) models. Comparison of the survival curve P values as derived from the Mantel-Haenszel test using the Kaplan-Meier estimate of survival (1); and the likelihood ratio test using the Cox proportional hazards regression model (2). P values of less than 0.05, using both models, are considered to indicate significant differences. (1) Kaplan, E., and P. Meier. 1958. Nonparametric estimation from incomplete observations. J. Am. Stat. Assoc. 53:457–481, 562–563. (2) Cox, D. R. 1972. Regression models and life tables. J. Royal Stat. Soc. Ser. B 34:187–220. (0.44 MB PDF) [file pone.0001356.s008.pdf]

**Inoculation method**	**Strains**	**Kaplan-Meier (p)**	**Cox proportional hazards model (p)**
Thoracic needle pricking	Canton-S vs. $act88F^{KM129}$	9.14^{-04}	1.88^{-03}
	Canton-S vs. hdp^{2}	2.42^{-05}	6.22^{-05}
	Canton-S vs. hep^{1}	5.3^{-05}	2.04^{-04}
	Canton-S vs. $Tm2^{3}$	2.33^{-3}	3.07^{-3}
	TpnC41C RNAi vs. *Yuri* RNAi	1.51^{-03}	1.59^{-03}
	$Gst2^{04227/06253}$ vs $Gst2^{06253/+}$	1.9^{-07}	5.33^{-07}
	$Gst2^{04227/06253}$ vs $Gst2^{04227/+}$	1.79^{-09}	1.03^{-08}
	w^{1118} vs $Gst2^{GS2160}$	5.71^{-03}	6.56^{-03}
	$Gst2^{GS2160}$ vs $Gst2^{GS2160}$;*dMef2-GAL4*	0.0424	0.0456
Abdominal needle pricking	Canton-S vs. $act88F^{KM129}$	0.981	0.954
	Canton-S vs. hdp^{2}	0.615	0.606
	Canton-S vs. hep^{1}	0.298	0.233
	Canton-S vs. $Tm2^{3}$	0.243	0.225
	TpnC41C RNAi vs. *Yuri* RNAi	0.801	0.788
Injector pumping (thorax)	Canton-S vs. hep^{1}	0.0775	0.0426
